# The Zittman Treatment of Syphilis

**Published:** 1909-07-17

**Authors:** 


### THE ZITTMAN TREATMENT OF SYPHILIS.

The essence of the treatment of syphilis advocated by Zittman appears to be prolonged diaphoresis and diuresis, brought about by drinking large quantities of highly-spiced liquids and by being kept in a room at a high temperature. It has been employed for certain cases at Netley since 1904, and Major Lawson gives an account both of the treatment and of its results in Vol. xii. of the "Journal of the Royal Army Medical Corps." It has been contended that it is particularly useful for patients who have already undergone a course of mercurial treatment, but who are, nevertheless, uncured; it is argued that the Zittman treatment assists in setting free the mercury that is already stored up in the bones and elsewhere in these cases. It is, therefore, to be employed in later rather than in early syphilis. Patients suffering from severe rashes, rupia, ulceration of the skin, bone affections, gummata, destructive lesions of the nose, and syphilitic rheumatic pains in the limbs and joints appear to benefit much, whereas affections of the mucous membranes do not seem to yield to this treatment in the same way.

Major Lawson reports that, notwithstanding the considerable discomforts that attend it, the course is very popular amongst the soldiers, who have the greatest belief in it and frequently ask to be put down for a second course. In many cases there is a loss of weight at first, but during the six weeks afterwards this loss becomes a gain. The chief difficulty in the treatment is to keep the room at the high temperature and yet ventilate it sufficiently.

The course lasts fifteen days. On the evening before it is begun the patient is given two pills according to the following prescription:—

| | |
|---|---|
| ℞ Hydrargyri subchloridi ... ... ... | gr. ii. |
| Extracti Colocynthi compositi ... ... | gr. v. |
| Extracti Hyoscyami ... ... ... | gr. ij. |

Misce. Fiat massa. Divide in pilulas duas.

He is ordered a free diet from which Zittman recommends that sugar and spices should be excluded, though this is not found to be necessary at Netley. He is kept in bed except for an hour in the evening, and the temperature of the room is maintained at 80° F. at least—better still at 84° F. or 85° F.

For the first four days the patient drinks half a pint of the following decoction as hot as possible, at 9, 10, 11 a.m., and 12 noon:—

Decoction No. 1.

| | |
|---|---|
| Rad. sarsæ contus. ... ... ... ... | ʒiv. |
| Sem. anisi contus. ... ... ... ... | gr. lxxx. |
| Sem. fœnic. contus. ... ... ... | gr. lxxx. |
| Foliæ sennæ ... ... ... ... | ʒj. |
| Rad. glycyrrh. contus. ... ... ... | ʒiv. |
| Add in a linen bag— | |
| Sacch. alb. ... ... ... ... ... | gr. lxxx. |
| Alum. sulph. ... ... ... ... | gr. lxxx. |
| Hydrarg. subchlor. ... ... ... | gr. lxxx. |
| Hyd. bisulph. rub. ... ... ... | gr. xx. |
| Aquæ ... ... ... ... ... | gals. iij. |

Boil down to one gallon and strain.

On the same day, at 3, 4, 5, and 6 p.m., he drinks half a pint of the following decoction cold:—

Decoction No. 2.

To the dregs of No. 1 Decoction add:

| | |
|---|---|
| Rad. sarsæ contus... ... ... ... | ℥ij. |
| Cort. limonis contus. ... ... ... | ʒj. |
| Sem. cardam. contus. ... ... ... | ʒj. |
| Rad. glycyrrh. contus. ... ... ... | ʒj. |
| Aquæ ... ... ... ... ... | gals. iij. |

Boil down to 1 gallon and strain.

On the fifth day the patient gets up, and in the evening he has two more pills. The treatment is repeated till the fifteenth day, when it is discontinued, and the patient returns to a ward at the usual temperature.

If diarrhœa results it will be found that the straining has not been carefully attended to, calomel being thereby allowed to pass into the decoction. As a matter of fact, calomel is practically insoluble, and the red bisulphide almost so; consequently the decoction contains next to no mercury, and equally good results have been reported when all mercury has been omitted from Decoction No. 1.

